# Is the NASA Lean Test a suitable tool to diagnose cardiovascular autonomic disorders?

**DOI:** 10.1007/s10286-024-01097-2

**Published:** 2025-01-09

**Authors:** Yvonne Teuschl, Alessandra Fanciulli, Anne Pavy-Le Traon, Mario Habek, Roland D. Thijs, Antun R. Pavelic, Walter Struhal

**Affiliations:** 1 Department for Clinical Neurosciences and Preventive Medicine, University for Continuing Education Krems, Dr. Karl-Dorrek-Straße 30, 3500 Krems, Austria; 2 Department of Neurology, Innsbruck Medical University, Anichstrasse 35, 6020 Innsbruck, Austria; 3 Department of Neurology, University Hospital of Toulouse, Toulouse, France; 4 Department of Neurology, University Hospital Center Zagreb, Zagreb, Croatia; 5 School of Medicine, University of Zagreb, Zagreb, Croatia; 6 Department of Neurology, Leiden University Medical Centre, Leiden, Netherlands; 7 Stichting Epilepsie Instellingen Nederland (SEIN), Heemstede, The Netherlands; 8 UCL Queen Square Institute of Neurology, University College London, London, UK; 9 Department of Neurology, University Hospital Tulln, Alter Ziegelweg 10, 3430 Tulln, Austria; 10 Karl Landsteiner University of Health Sciences, Dr. Karl-Dorrek-Straße 30, 3500 Krems, Austria

**Keywords:** Autonomic nervous system, Passive stand test, NASA lean test, Orthostatic intolerance

Dear editors,

In recent years, the National Aeronautics and Space Administration (NASA) Lean Test (NLT) was introduced as point-of-care assessment for orthostatic intolerance in the context of myalgic encephalomyelitis/chronic fatigue syndrome (ME/CFS) [1]. The NLT is an orthostatic challenge, during which heart rate and blood pressure are recorded after the subject has been resting in a supine position for 10 min, then moves to a standing position and leans against a wall for further 10 min [1]. During the coronavirus disease 2019 (COVID-19) pandemic, when access to medical care was restricted, telemedicine and self-management became increasingly important [2]. In this scenario, the NLT gained momentum for the identification of cardiovascular autonomic disorders because of its simplicity and ubiquitous availability [3]. This went so far that individuals suffering from orthostatic intolerance sometimes diagnosed themselves with postural orthostatic tachycardia syndrome (POTS) on the basis of a self-administered NLT before a structured cardiovascular autonomic assessment could be performed. Diagnoses in such settings carried high inaccuracy rates, because recording did not follow clinical standards, settings were inappropriate, and devices unreliable.

Patients with post-COVID-19 often experience fatigue and cardiovascular autonomic disturbances [4], and have been compared with those suffering from ME/CFS. Accordingly, a small study proposed to use the NLT as an office-based test to evaluate orthostatic intolerance in patients with post-COVID-19, as well as in those with ME/CFS [5]. Despite the rapid dissemination of the NLT, validation studies comparing the diagnostic accuracy of the NLT with current gold standards, i.e., tilt table testing or an active standing test for the detection of orthostatic hypotension (OH) or POTS, are lacking.

Standard diagnoses for OH and POTS have been defined by consensus on the basis of hemodynamic criteria for head-up tilt or active standing tests [6]. However, hemodynamic criteria alone do not make the diagnosis. For a final diagnosis of a cardiovascular autonomic syndrome (e.g., POTS) more criteria have to be fulfilled [7]. During a tilt table test, movement is restricted and subjects are passively moved into a 70° (60–80°) upright position on the basis of the European Consensus Criteria [6]. In contrast, during active standing the skeletal muscle pump is active, which increases the venous return to the heart, in turn leading to a different cardiovascular response compared with passive tilt [8]. Applying hemodynamic diagnostic criteria established for head-up tilt or active standing test to the NLT [1, 3, 5] might be problematic because of the leaning position itself.

The NLT is based on physiological tests that have been performed in the past by NASA in small samples of healthy young men or astronauts. These experiments investigated orthostatic tolerance after exposure to different physiological challenges, such as heat, exercise, and prolonged bed rest, as well as interventions to prevent cardiovascular deconditioning after being exposed to microgravity during space flight [9–11]. Leaning against a wall was proposed as an alternative to the passive tilt table test to measure orthostatic tolerance [9, 11]. This was tested in one experiment by comparing test-induced heart rate alterations of five men after three tests (70° tilt, lower body negative pressure, and a lean test) before and after 2 weeks of bed rest [9]. Prolonged bed rest is commonly used to simulate microgravity. The test-induced heart rate changes were larger in the lean test than in the 70° tilt test; nevertheless, all three tests were judged as being suited to measure an orthostatic response [9]. In the abovementioned experimental context, lean tests were used to investigate normal physiological reactions, and not for the identification of orthostatic disorders. In space flight, a free-standing test might be challenging for astronauts returning to normal gravity on the basis of complex physiologic re-adaptation to gravity including the cardiovascular, neurovestibular, musculoskeletal, and endocrine systems. Furthermore, tilt table testing may not be possible at the landing area of a space flight. At the present time, however, NASA refrains from using lean tests and is currently using a standard 10 min active standing test instead.

Recent publications using the NLT for orthostatic testing in conditions of chronic fatigue [3, 5] refer to these papers from the 1970s [9–11]. Citing the old NASA experiments, lean tests were regarded as a substitute for passive tilt table tests, and diagnosis criteria for OH and POTS were used accordingly [1, 3, 5]. Despite the definition of “leaning” provided in the instructions of the NLT (only shoulder blades should contact the wall with the heels six inches (15 cm) away from the wall, and patients should avoid moving, tensing, or shifting their weight; it might be difficult to standardize body posture in a way to preclude the use of the calf muscle pump. A lean test may thus be interpreted as a mixture between active standing and passive tilt.

In the past, a large variety of test protocols and measurement points have further complicated the choice of an adequate test and its interpretation. In the last years, however, consensus has been reached about diagnostic criteria and experts of the autonomic nervous system field have provided recommendations for the administration and interpretation of active standing tests, as well as for tilt table tests [6, 12]. There are currently no structured data regarding the validity of a leaning test for the clinical autonomic diagnosis of orthostatic disorders. It is therefore preferable to use active standing tests. Active standing tests can be used to screen for POTS. However, passive tilt and active standing are distinct tests [12]. If the objective of the evaluation is to diagnose clinical autonomic involvement in a patient, it is recommended that standardized test protocols, in accordance with international consensus (e.g. [6]), be applied.

The NLT may currently not be used as a diagnostic tool. In general, results of provocative cardiovascular tests should be carefully recorded and interpreted by specialists, and not by patients themselves. Controlling for confounding factors and measurement by trained persons had, in the past, been developed as an important quality assurance for providing reliable hemodynamic test results. The NLT might seem an appealing alternative to time-consuming and resource-intensive testing; however, on the basis of the current available data, it has to be considered as an improper shortcut to cardiovascular autonomic diagnoses.
